# High‐salt intake reduces renal tissue levels of inflammatory cytokines in mice

**DOI:** 10.14814/phy2.14621

**Published:** 2020-12-20

**Authors:** Purnima Singh, Roxan Stephenson, Alexander Castillo, Dewan S. A. Majid

**Affiliations:** ^1^ Department of Physiology, Hypertension and Renal Centre of Excellence Tulane University School of Medicine New Orleans LA USA

**Keywords:** endothelial nitric oxide synthase, high‐salt intake, IL‐10, IL‐6, nitric oxide, TNF‐α

## Abstract

High salt (HS) intake is usually considered as an aggravating factor to induce inflammatory renal injury. However, the changes in the renal levels of inflammatory cytokines during HS intake is not yet clearly defined. We hypothesize that HS increases renal levels of tumor necrosis factor‐alpha (TNF‐α) and interleukin‐6 (IL‐6) but decreases interleukin‐10 (IL‐10; anti‐inflammatory cytokine) and these responses exacerbate in NO deficient conditions. Both wild‐type (WT) and endothelial NO synthase knockout (eNOSKO) mice (~8 weeks old, *n* = 6 in each group) were given normal‐salt (NS; 0.3% NaCl) and HS (4% NaCl) containing diets for 2 weeks. Systolic blood pressure (SBP) was determined by tail‐cuff plethysmography and urine collections were made using metabolic cages. Basal SBP was higher in eNOSKO than WT mice (131 ± 7 vs 117 ± 3 mmHg; *p* < .05). HS intake for 2 weeks increased SBP in eNOSKO (161 ± 5 mmHg) but not in WT mice. In NS groups, the cytokine levels in renal tissues (measured using ELISA kits and expressed in pg/mg protein) were significantly higher in eNOSKO than WT mice (TNF‐α, 624 ± 67 vs. 325 ± 73; IL‐6, 619 ± 106 vs. 166 ± 61; IL‐10, 6,087 ± 567 vs. 3,929 ± 378). Interestingly, these cytokine levels in HS groups were significantly less both in WT (TNF‐α, 114 ± 17; IL‐6, 81 ± 14; IL‐10, 865 ± 130) and eNOSKO (TNF‐α, 115 ± 18; IL‐6, 56 ± 7; IL‐10, 882 ± 141) mice. These findings indicate that HS induces downregulation of cytokines in the kidney. Such HS‐induced reduction in cytokines, particularly TNF‐α (a natriuretic agent), would facilitate more salt‐retention, and thus, leading to salt‐sensitive hypertension in NO deficient conditions.

## Graphical abstract

This study examined the renal levels of inflammatory cytokines during high salt (HS; 4% NaCl in diet) intake for 2 weeks in mice. The findings indicate that HS induces a downregulation of cytokines in the kidney. Such HS induced reduction in cytokines, particularly TNF‐α (a natriuretic agent), would facilitate more salt‐retention and thus, leading to salt‐sensitive hypertension in NO deficient conditions.

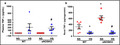

## 1 INTRODUCTION

It is generally perceived that a high‐salt (HS) diet is associated with many cardiovascular and metabolic disorders such as hypertension and exacerbation of inflammation. Evidences are no less to suggest that a HS diet is associated with higher biomarkers of inflammation. Many recent studies have also contributed to the fact that hypertension is a chronic inflammatory disease condition with an imbalance between pro‐inflammatory and anti‐inflammatory cytokines. Such imbalance is induced by dysregulation of the renin‐angiotensin system (RAS) and oxidative stress, which are associated with increased dietary salt intake and aging (Das, 2006; Guzik et al., 2007; Mattson, 2014; Rodriguez‐Iturbe et al., 2004). Not only as an inducer of oxidative stress, but angiotensin II (AngII) is also known to induce increased production of inflammatory cytokines such as tumor necrosis factor‐alpha (TNF‐α) and interleukin (IL‐6) (Funakoshi et al., 1999; Han et al., 1999; Ruiz‐Ortega et al., 2002; Sanz‐Rosa et al., 2005). In experiments with rodent models, chronic intake of the HS diet does not alter systemic blood pressure (BP) (Kopkan et al., 2010; Lara et al., 2012). However, HS diet fed to rodents‐treated chronically with AngII or a NO synthase (NOS) inhibitor, nitro‐L‐arginine‐methyl ester (L‐NAME) or in endothelial NOS knockout (eNOSKO) mice exaggerated the hypertensive response, and renal inflammatory tissue injury (Francois et al., 2008; Kopkan et al., 2010; Kopkan & Majid, 2005; Nakamura et al., 2011; Singh et al., 2013, 2017). HS intake in eNOSKO mice has been shown to augment hypertension and renal injury associated with increased oxidative stress and the production of AngII (Nakamura et al., 2011). These results indicate that HS diet influences the production of inflammatory cytokines in the condition of increased oxidative stress or in enhanced RAS activity. We have reported previously that acute inhibition of NOS by systemic infusion of L‐NAME in mice increased the plasma and renal level of TNF‐α (Shahid et al., 2010; Singh et al., 2014), with increased infiltration of macrophages in the kidney (Islam et al., 2011). Such systemic NOS inhibition by acute L‐NAME infusion in mice reduced the level of the anti‐inflammatory cytokine, interleukin‐10 (IL‐10) in the plasma and renal tissue (Singh et al., 2014). However, the changes in the inflammatory cytokine levels in response to chronic HS intake in normal conditions and the condition of chronic NO deficiency are not yet clearly determined.

We hypothesize that HS intake enhances renal levels of pro‐inflammatory and reduces anti‐inflammatory cytokines, and these responses exacerbate in the condition of NO deficiency. To examine this hypothesis, we aimed to assess the changes in the status of inflammatory cytokines, particularly in the kidney during intake of HS diet, and to determine how these cytokines are affected by chronic NO deficiency. In this study, we measured the renal as well as plasma levels of pro‐ (IL‐6 and TNF‐α) and anti‐ (IL‐10) inflammatory cytokines in wild‐type (WT) and eNOSKO mice which were fed normal‐salt (NS; 0.3% NaCl) and HS (4% NaCl) containing diets for 2 weeks.

## 2 MATERIALS AND METHODS

The experiments were performed in accordance with the guidelines and practices established by the Tulane University Animal Care and Use Committee. Male eNOSKO mice (B6.129P2‐Nos3tm1Unc/J; stock no: 002684) mice and their genetic background strain of WT (C57BL/6J; stock no: 002684) mice (Jackson Laboratory, Bar Harbor, ME) considered as control were used in this study. These mice were housed in a temperature‐ and light‐controlled room and allowed free access to a standard diet (Ralston‐Purina, St. Louis, MO) and tap water. The mice (8–9 week of age; ∼20–22 g body weight) were randomly divided into different groups depending on the intake of NS diet (a standard diet containing 0.3% NaCl) and HS diet (HS, 4% NaCl; Harlan‐Teklad, Madison, WI), respectively, comprised of six animals in each group. The groups are as follows:

1. WT‐NS: Wild‐type mice fed the NS diet
2. WT‐HS: Wild‐type mice fed the HS diet
3. eNOSKO‐NS: eNOSKO mice fed the NS diet
4. eNOSKO‐HS: eNOSKO mice fed the HS diet

### 2.1 BP monitoring in conscious mice

The systolic BP (SBP) was measured in the experimental mice using the noninvasive tail‐cuff plethysmography technique (Visitech Systems, Apex, NC). The mice were monitored for 7–10 days on a standard laboratory diet, and basal BP was recorded before being switched to a high‐salt diet (only for WT‐HS and eNOSKO‐HS mice) for the remainder of the study. In these groups of mice, BP was measured at the same time of the day (11 a.m. to 1 p.m.) to avoid the influence of the circadian cycle, and the value of SBP was obtained by estimating the average reading of 10 measurements for a single trial. The mice were trained for tail‐cuff BP measurements 7–10 days before starting the experiments. After 2–3 days of acclimatization to the tail‐cuff device, the basal SBP was measured in all the mice on standard laboratory diet before the start of HS diet and considered as Day 0 value. Then, the animals of HS group were switched to HS diet for the remainder of the study (subsequently SBP was measured on days 3, 6, 9, and 12 of NS/HS diets) on a 12:12‐hr light‐dark cycle and received food and water ad libitum throughout the study.

### 2.2 Animal treatment

After the recording of resting SBP, mice were fed with either a NS (0.3% NaCl) or HS (4% NaCl) diet for 2 weeks to evaluate the responses to HS alone in WT and eNOSKO mice. Both these diets contained approximately similar concentration of other nutrients (19% protein, 5% fat, 3% crude fiber, 0.86% Ca, 0.64% P, 0.72% K, and 0.15% Mg). During the experimental period, the mice were allowed drinking freely from the water bottles attached to the cages. Food and water intake, along with bodyweight, of each mouse was measured during the experimental period.

### 2.3 Collection and analysis of urine and blood samples

Twenty‐four‐hour urine samples were collected from conscious mice using metabolic cages on the basal day (Day 0) and on the last day (Day 13) to evaluate the excretory parameters with or without HS intake in these mice. All the groups of mice were placed in metabolic cages and urine, and the necessary parameters were collected at the same time. Animals were housed individually in metabolic cages, and urine was collected for 24 hr into sterile tubes. Urine volumes were determined from each urine collection, and samples were centrifuged (3,000 rpm/5 min; 4°C) and preserved for determining urinary excretion of sodium and potassium. Urinary excretion of sodium and potassium was assessed by flame photometry (Singh et al., 2013, 2017).

At the end of the experimental period, all the animals were euthanized, and blood was collected. Plasma was immediately separated from the blood and stored at −80ºC for estimation of pro‐ (IL‐6 & TNF‐α) and anti‐ (IL‐10) inflammatory cytokines as conducted earlier (Singh et al., 2014, 2017). The kidneys were also removed. One kidney was fixed in 10% of formalin solution and paraffin‐embedded for evaluation of the renal injury. The other kidney was processed to measure the levels of inflammatory cytokines. A 100 mg kidney comprising of both cortical and medullary regions was homogenized in sterile phosphate‐buffered saline containing protease inhibitor at 4°C. The kidney homogenates were centrifuged at 9,000 g for 10 min at 4°C. The supernatants were transferred to clean micro‐centrifuge tubes and stored at −80°C until analyzed.

### 2.4 Enzyme‐linked immunosorbent assay for IL‐10, IL‐6, and TNF‐α level

Levels of IL‐10, IL‐6, and TNF‐α in plasma and supernatants from kidney tissue homogenates were measured using ELISA Ready‐SET‐go kits (eBioscience, Inc., San Diego, CA). The detection levels of the kits (standard curve range) are as follows: IL‐10 kit (Catalog no. 88‐7105‐88), 32–4000 pg/ml; IL‐6 Kit (Catalog no. 88‐7064‐22), 4–500 pg/ml; TNF‐α Kit (Catalog no. 88‐7324‐86), 8–1000 pg/ml. The levels of cytokines in renal tissue were normalized by protein concentration (measured by Bio‐Rad detergent compatible protein assay method) (Cat. No. # 500‐0112; Bio‐Rad, Hercules, CA).

### 2.5 Evaluation of renal injury

The formalin‐fixed paraffin‐embedded kidney sections were used for analyzing glomerulosclerosis using periodic‐acid‐Schiff staining (Lara et al., 2012; Luna, 1992; Singh et al., 2013, 2017) and tubulointerstitial fibrosis using Gomori's trichrome staining (Gomori, 1950; Singh et al., 2013, 2017).

### 2.6 Estimation of glomerulosclerosis

The extent of glomerulosclerosis was evaluated quantitatively by automatic image analysis of each glomerulus using periodic‐acid‐Schiff stained renal sections (Mass Histology Service, Worcester, MA) (Lara et al., 2012; Luna, 1992; Singh et al., 2013, 2017). Twenty images from each kidney slide with at least one glomerulus per field were photographed using a Nikon Eclipse 50i microscope equipped with a Nikon DS Camera Head (DS Fi1) and DS camera control unit (DSU2). A dark purple color in the glomerulus was recognized as sclerosis. The percentage area covered by sclerosis in glomeruli in each field was analyzed using the Nikon NIS‐Elements software (version 2.34). The percentage of data obtained for each of the 20 images were averaged to obtain the percentage area of sclerosis for the entire slide.

### 2.7 Estimation of tubulointerstitial fibrosis

The extent of the interstitial collagen‐positive area (fibrosis) was evaluated quantitatively by automatic image analysis of the renal section occupied by interstitial tissue staining positively for collagen in Gomori's trichrome‐stained sections (Gomori, 1950; Singh et al., 2013, 2017). Formalin‐fixed paraffin‐embedded renal sections were stained with a plasmin stain (chromotrope 2R) and a connective tissue fiber stain (aniline blue) combined in a solution of phosphotungstic acid to which glacial acetic acid had been added. This stained the collagen blue, which indicates fibrosis. Slides were photographed as described above. The percentage area covered by collagen in each field was analyzed using the Nikon NIS‐Elements software (version 2.34). The percentage of data obtained for each of the 20 images were averaged to obtain the percentage area of fibrosis for the entire slide.

### 2.8 Statistical analysis

All results are expressed as means ± SE. Statistical analysis was performed using Sigma stat software (Systat Software, Chicago, IL). A comparison of the responses within the same group and between the groups was conducted using the repeated measures ANOVA and Dunnett multiple comparisons test. *p* ≤ .05 is considered as significant.

## 3 RESULTS

### 3.1 BP responses

**FIGURE 1:**
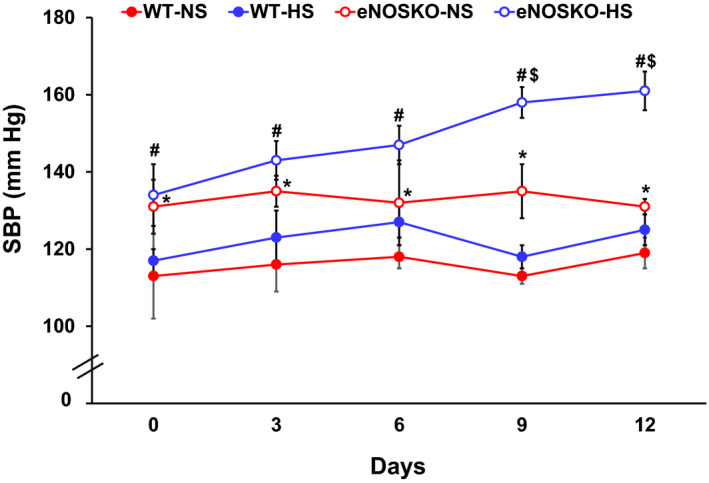
Systolic blood pressure (SBP) in WT and eNOSKO mice fed with either normal (NS) or high‐salt (HS) diets. NS or HS diets were given for 2 weeks. The values at basal day before start of HS diet represent basal values at the start of the experiment and considered as Day 0 value. *N* = 6/group. Values are Mean ± *SEM*; *p < .05 versus WT‐NS; ^#^ *p* < .05 versus WT‐HS‐NS; $*p* < .05 versus WT‐HS

The basal (Day 0) level of SBP was higher in eNOSKO than WT mice (131 ± 7 vs. 117 ± 3, mmHg; *p* < .05) as illustrated in Figure 1a. HS intake alone for 2 weeks caused a significant increase in SBP in eNOSKO but not in WT mice (161 ± 5 vs. 125 ± 4, mmHg; *p* < .05; Figure 1a).

### 3.2 Renal excretory responses

The basal rate of urine flow and sodium excretion was similar in eNOSKO and WT mice fed with the NS diet. The intake of HS containing diets for nearly 2 weeks increased the rate of water intake, urine flow, and sodium excretion in both the groups similarly.

The values for body weight, food, and water intake, and excretory parameters in urine collected from mice on day 0 and day 13 (last day) of the experimental period are given in Table 1. The metabolic data that is obtained in the present study is similar as was reported earlier from our laboratory (Kopkan et al., 2010). However, it should be noted that these metabolic data have been collected for only separate days within 2 weeks period of study without collecting the fecal matter and other measurements of insensible loss of salt and water. Thus, a comprehensive assessment in sodium and water balance during the whole experimental period is not possible from these collected parameters from the metabolic cages.

**TABLE 1:** Water intake and urinary excretory parameters in different groups of mice on Day 0 and Day 13 of the experimental period with NS/HS intake

		WT		eNOSKO	
		NS	HS	NS	HS
Body weight (g)	Day 0	20.70 ± 0.20	20.60 ± 0.20	20.70 ± 0.40	20.30 ± 0.60
	Day 13	22.42 ± 0.18	22.50 ± 0.50	21.00 ± 0.50	21.20 ± 0.60
Food intake (g/24 hr)	Day 0	0.80 ± 0.30	0.60 ± 0.00	0.70 ± 0.20	0.60 ± 0.10
	Day 13	0.62 ± 0.14	1.55 ± 0.38*	0.80 ± 0.20	1.6 ± 0.30^#^
Water intake (ml/24 hr)	Day 0	2.083 ± 0.400	1.300 ± 0.380	1.800 ± 0.307	1.833 ± 0.307
	Day 13	1.917 ± 0.436	5.700 ± 0.464*	1.267 ± 0.410	4.300 ± 0.606^#^
Urine flow (ml/24 hr)	Day 0	1.697 ± 0.150	1.340 ± 0.094	1.410 ± 0.208	1.408 ± 0.208
	Day 13	1.382 ± 0.102	2.704 ± 0.162*	1.058 ± 0.083	2.829 ± 0.469^#^
Sodium excretion (mM/24 hr)	Day 0	0.176 ± 0.017	0.157 ± 0.014	0.201 ± 0.008	0.126 ± 0.010
	Day 13	0.169 ± 0.013	1.325 ± 0.153*	0.152 ± 0.013	1.100 ± 0.222^#^
Potassium excretion (mM/24 hr)	Day 0	0.211 ± 0.029	0.243 ± 0.015	0.282 ± 0.031	0.212 ± 0.058
	Day 13	0.303 ± 0.051	0.252 ± 0.042	0.273 ± 0.008	0.132 ± 0.019

Note

Values are Mean ± *SEM*; *n* = 6/group.

Abbreviations: eNOSKO, endothelial nitric oxide synthase knockout mice; HS, High‐salt (4% NaCl) containing diet; NS, normal‐salt (0.3% NaCl) containing diet; WT, WT mice.

* *p* < .05 versus WT‐NS.

^#^ *p* < .05 versus eNOSKO‐NS.

### 3.3 Inflammatory cytokine levels

#### 3.3.1 TNF‐α

**FIGURE 2:**
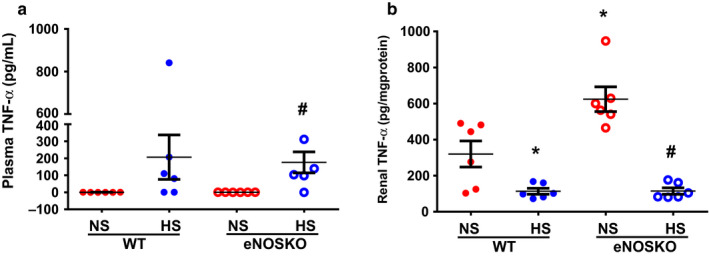
Tumor necrosis factor‐alpha (TNF‐α) levels (a – in plasma; b – in renal tissues) in WT and eNOSKO mice fed on either normal (NS) or high‐salt (HS) diets. Values are Mean ± *SEM*; *n* = 6/group. *p < .05 versus WT‐NS; #*p* < .05 versus eNOSKO‐NS; UD = undetected level

Figure 2 depicts the plasma and renal tissue levels of TNF‐α (Figure 2a and b) at the end of the experimental period. The TNF‐α concentration in the plasma was undetected in NS intake groups, both WT and eNOSKO mice. However, in the HS intake groups of mice, the plasma TNF‐α level was detected with high variability in WT, 202 ± 146 pg/ml and eNOSKO, 175 ± 68 pg/ml) mice. In the NS intake groups, the TNF‐α level in renal tissue was higher in the eNOSKO than WT mice (624 ± 67 vs. 325 ± 73, pg/mg protein; *p* < .05). However, in the HS intake groups, renal tissue TNF‐α levels were significantly lower in WT (114 ± 17 pg/mg protein) and eNOSKO (115 ± 18 pg/mg protein) mice compared to NS intake groups.

#### 3.3.2 IL‐6

**FIGURE 3:**
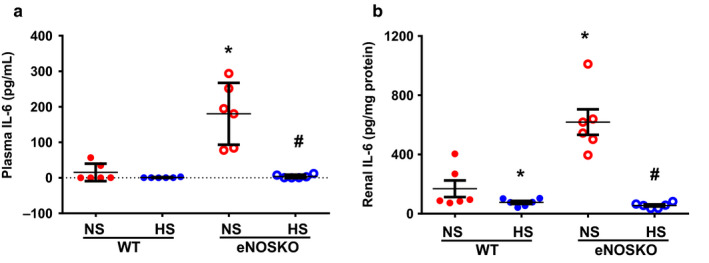
Interleukin‐6 (IL‐6) levels (a – in plasma; b – in renal tissues) in WT and eNOSKO mice fed on either normal (NS) or high‐salt (HS) diets. Values are Mean ± *SEM*; *n* = 6/group. *p < .05 versus WT‐NS; ^#^ *p* < .05 versus eNOSKO‐NS; UD = undetected level

Figure 3 depicts the plasma and renal tissue levels of IL‐6 (Figure 3a and b). In the NS intake groups, IL‐6 concentration in the plasma (180 ± 44 vs. 13 ± 11 pg/ml; *p* < .05) and in the kidney (619 ± 106 vs. 166 ± 61 pg/mg protein; *p* < .05) in eNOSKO was higher than WT mice. However, plasma IL‐6 level in HS intake groups was undetected in both WT and eNOSKO mice. Renal tissue IL‐6 level was also lower significantly in HS intake groups in both eNOSKO (56 ± 7 pg/mg protein) and WT (81 ± 14 pg/mg protein) mice compared to the NS intake groups.

#### 3.3.3 IL‐10

**FIGURE 4:**
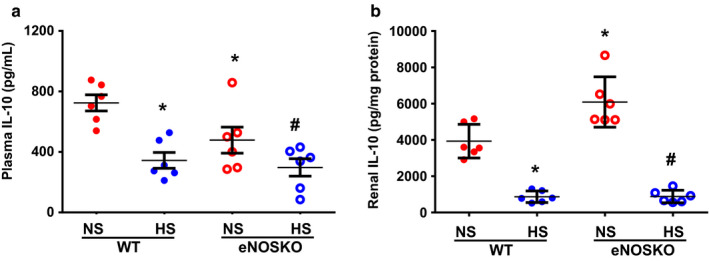
Interleukin‐10 (IL‐10) levels (a – in plasma; b – in renal tissues) in WT and eNOSKO mice fed on either normal (NS) or high‐salt (HS) diets. Values are Mean ± *SEM*; *n* = 6/group. *p < .05 versus WT‐NS; ^#^ *p* < .05 versus eNOSKO‐NS

Figure 4 depicts the plasma and renal tissue levels of IL‐10 (Figure 4a and b). In NS intake groups, the plasma level of IL‐10 in eNOSKO was lower than WT mice (510 ± 125 vs. 701 ± 54 pg/ml; *p* < .05). However, the renal tissue IL‐10 level was higher in the eNOSKO than WT mice (6,087 ± 567 vs. 3,929 ± 378, pg/mg protein; *p* < .05). In HS intake group, plasma and renal tissue levels of IL‐10 were significantly lower in both eNOSKO (327 ± 67 pg/ml; 882 ± 141 pg/mg protein) and WT (344 ± 52 pg/ml; 865 ± 130 pg/mg protein) mice.

### 3.4 Renal injury parameters

#### 3.4.1 Glomerulosclerosis

**FIGURE 5:**
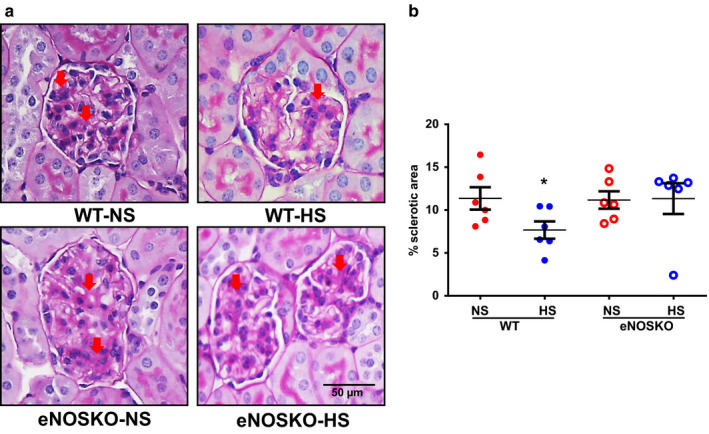
Renal injury as indicated by glomerular sclerosis responses WT and eNOSKO mice fed with either normal (NS) or high‐salt (HS) diets. (a) illustrates the representative photomicrographs, and (b) illustrates the mean values of the percent areas of glomerular sclerosis in the renal tissues, respectively. *p < .05 versus WT‐NS

The representative images of the periodic‐acid‐Schiff stained kidney sections providing the extent of glomerular sclerosis are given in Figure 5a and the percent of the sclerotic areas are illustrated in Figure 5b. Though we observed contracted and thrombosed glomeruli in the NS‐fed eNOSKO but not WT mice, there was no significant difference in the percent sclerotic area between WT‐NS and eNOSKO‐NS mice (11.4 ± 1% and 11.2 ± 1%). However, the percent sclerotic area was lower in WT‐HS compared to WT‐NS mice (7.7 ± 1% vs. 11.4 ± 1%; *p* < .05). On the contrary, the percent of sclerotic areas were similar in eNOSKO‐NS and eNOSKO‐HS mice (11.2 ± 1% and 11.3 ± 1%).

#### 3.4.2 Tubulointerstitial fibrosis

**FIGURE 6:**
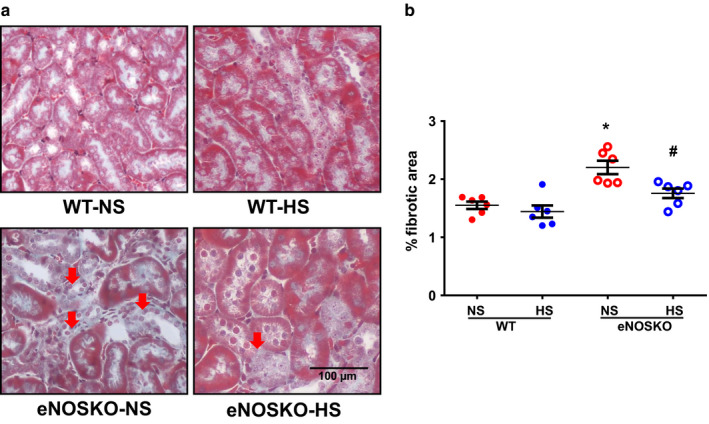
Renal injury as indicated by interstitial fibrosis responses WT and eNOSKO mice fed with either normal (NS) or high‐salt (HS) diets. (a) illustrates the representative photomicrographs, and (b) illustrates the mean values of the percent areas of interstitial fibrosis in the renal tissues, respectively. Values are Mean ± *SEM*; *n* = 6/group. *p < .05 versus WT‐NS; ^#^ *p* < .05 versus eNOSKO‐NS

The representative images of Gomori's trichrome‐stained sections providing the extent of renal interstitial fibrosis are given in Figure 6a, and the percent of the fibrotic areas are illustrated in Figure 6b. Overall, the fibrotic values in the renal interstitium are quantitatively lower in these mice models. In WT mice, the fibrotic area in the renal interstitium was similar in both NS and HS intake groups. Nevertheless, as expected, the fibrotic area was significantly higher in eNOSKO‐NS compared to WT‐NS mice (2.2 ± 0.2% vs. 1.5 ± 0.2%; *p* < .05). However, the fibrotic area in the renal interstitium was lower in eNOSKO‐HS than eNOSKO‐NS mice (1.8 ± 0.1% vs. 2.2 ± 0.2%; *p* < .05).

## 4 DISCUSSION

Although chronic NO deficiency induces an enhancement in inflammatory cytokines, the results of the present study indicate that HS intake in mice, at least for 2 weeks, generally suppresses the levels of both pro‐ and anti‐inflammatory cytokines, particularly in the kidney. The basal plasma level of TNF‐α was mostly undetected in both WT and eNOSKO mice that were fed NS diets. However, a significant level of this cytokine was noted in both strains fed HS diet for 2 weeks period. Therefore, it indicates that HS intake stimulates the systemic production of TNF‐α, as indicated in other studies (Costa et al., 2012; Liu et al., 2012; Yilmaz et al., 2012; Zhu et al., 2014). However, it is interesting to note that the renal levels of TNF‐α are significantly lower in the HS intake groups of WT and eNOSKO mice compared to that in the NS intake groups. Such HS‐induced reduction was also noted in other cytokines such as IL‐6 and IL‐10 in the kidney. Although the renal levels of IL‐6 and IL‐10 were higher in eNOSKO‐NS mice than those in WT‐NS mice, their plasma levels were variable. Compared to WT‐NS mice, plasma levels of IL‐6 were higher, IL‐10 was lower, but the TNF‐α level was not different in eNOSKO‐NS mice. Anyway, the higher renal levels of pro‐inflammatory cytokines in eNOSKO‐NS mice is somewhat expected. NO is a signaling molecule that usually plays an anti‐inflammatory function under normal physiological conditions (Harrison, 1997; Moncada et al., 1991). NO deficiency is generally known to play a key role in inducing the inflammatory process (Liu & Huang, 2008). However, the level of a known anti‐inflammatory cytokine, IL‐10, is also higher in the renal tissue of eNOSKO‐NS mice compared to that in WT‐NS mice. This could be due to the fact IL‐10 does not always act as an anti‐inflammatory agent but can also act as a pro‐inflammatory cytokine in certain conditions linked to elevated RAS, as demonstrated earlier from our laboratory (Singh et al., 2017).

Although it is generally perceived that a HS diet is usually associated with the induction of higher biomarkers of inflammation, most of these findings are observed in population studies in which HS diets are often coded for subjective food associated with more fat and protein‐rich diets, which could be skewing some of these results (Zhu et al., 2014). An interesting recent study also suggested that consumption of refined salt, but not natural sea‐salt, induces hypertension and cause abnormal kidney pathology in Dahl salt‐sensitive rats (Lee et al., 2017). Thus, there exists a complex relationship between salt intake and immunity development in the human body. The present study is the first among the studies using animal models to demonstrate the responses to a more controlled intake of HS diets on the levels of some inflammatory cytokines in plasma and in the kidney. The observed reduction in the renal production of cytokines during HS intake could be related to the suppression of RAS due to HS intake (Drenjančević‐Perić et al., 2011).

It is noted that a considerably high level of plasma IL‐10 was detected in NS‐fed WT mice, but its level was only slightly lower in NS‐fed eNOSKO mice. On the contrary, a marked reduction of IL‐10 was observed in response to acute systemic NOS inhibition in mice, as reported in our earlier study (Singh et al., 2014). However, IL‐10 levels in plasma remained lower in WT and eNOSKO mice during HS intake in the present study. These data indicate that chronic HS intake generally suppresses the levels of both anti‐ and pro‐inflammatory cytokines, particularly in the kidney. In eNOSKO mice, renal tissue nitrotyrosine level is lower compared with that in WT mice (Kopkan et al., 2010) indicating that peroxynitrite production is less in these eNOS deficient mice and this may help to enhance IL‐10 level in this strain of mice (Cook et al., 2003; Numanami et al., 2003). The higher plasma IL‐10 level could produce an inhibitory effect on the plasma level of TNF‐α in eNOSKO mice, as it has been shown that acute infusion of IL‐10 in mice reduces TNF‐α level in the kidney (Singh et al., 2014). In certain conditions, however, IL‐10 is shown to act as a pro‐inflammatory agent to induce renal injury, particularly during elevated angiotensin II in mice (Singh et al., 2017). Thus, it is possible that IL‐10 acts as a pro‐inflammatory cytokine in this eNOS gene knockout strain.

As usual, HS intake did not alter BP in WT but caused a significant increase in BP in eNOSKO mice (Kopkan et al., 2010) in which baseline BP was higher as compared to WT mice. As HS intake induces reductions in cytokine levels in both the strains of mice, such response in eNOSKO mice may not suggest any direct relationship between BP changes with the changes in the inflammatory cytokines. There is also no evidence available so far, that change in arterial pressure directly influences the production of TNF‐α but on the contrary, we and others have reported that intravenous infusion of human recombinant TNF‐α decreases BP in mice (Kramer et al., 1988; Shahid et al., 2008). In the present study, similar changes in plasma and renal levels of TNF‐α and other cytokines in WT and eNOSKO mice have been observed though BP increased only in the eNOSKO mice and not in WT mice. This observation would not be entirely surprising as many other interventional studies in humans (Luo et al., 2016) also suggested such a nonlinear relationship with inflammatory cytokines and hypertension during HS intake. In a randomized placebo‐controlled crossover study in untreated (pre)hypertensive subjects, it was also noted that a 4‐week period of salt intake did not affect inflammatory markers such as plasma levels of TNF‐α, IL‐1β, IL‐6, IL‐8, or MCP‐1) (Gijsbers et al., 2015). It was also noted that BP in humans was remained unchanged during low salt intake that resulted in an increased production of pro‐inflammatory cytokines like TNF‐α, IL‐6, or pro‐calcitonin (Mallamaci et al., 2013). Thus, a direct correlation between the changes in inflammatory cytokines and the BP changes is not yet proven experimentally.

Our previous studies in rodents (Kopkan et al., 2010; Kopkan & Majid, 2005; Singh et al., 2017) have demonstrated that endogenous NO production by eNOS minimizes salt‐sensitivity in response to the intake of HS diet. Studies in animals under normal conditions suggest that chronic HS intake alone causes no or minimal change in BP. However, HS intake induces salt sensitivity and hypertension when NO production is compromised in eNOSKO mice (Kopkan et al., 2010) or rats (Kopkan & Majid, 2005) chronically treated with a NOS inhibitor, L‐NAME. Our observation in the present study suggests that HS intake induces salt‐sensitive hypertension only in eNOSKO mice but not WT mice (Figure 1). This is most likely due to enhancement in tubular sodium reabsorption in the kidney that results from increased tissue superoxide level when NO production by eNOS activity is compromised (Kopkan et al., 2010). Chronic HS intake usually induces NO production by upregulating eNOS activity (Mattson & Higgins, 1996). Such an increase in NO level minimizes the tissue superoxide activity to maintain a condition of “oxidative balance” that facilitates normal sodium homeostasis, and thus, minimize the increases in BP. We have also demonstrated that hypertensive response to chronic HS intake in Il‐10 knockout mice is reduced due to enhancement in eNOS expression in these mice (Singh et al., 2017). An increase in plasma TNF‐α level during chronic HS intake also contributes in minimizing such enhancement in tubular sodium reabsorption, due to an increase in sodium load in WT mice despite a reduction in renal tissue level of TNF‐α. However, in the condition of oxidative imbalance due to NO deficiency, such a protective effect of plasma TNF‐α level is compromised, leading to enhanced sodium retention, and thus, caused salt sensitivity and hypertension in eNOSKO mice. We have previously demonstrated that an increase in plasma TNF‐α level induces a natriuretic response via activation of tubular TNF‐α receptor type 1 (TNFR1) (Castillo et al., 2012). Our recent preliminary data also demonstrate that TNFR1 protein expression is enhanced during chronic HS intake in WT mice but reduced in NO deficient condition either in eNOSKO mice (Majid et al., 2018) or in mice chronically treated with L‐NAME (Majid et al., 2017). Such a reduction in TNFR1 activity also contributes to salt sensitivity and hypertension during HS intake in eNOSKO mice. Thus, it is conceivable that eNOS has a significant role in minimizing the impact of chronic HS intake in WT mice.

Chronic HS intake, through “osmoreceptor function,” induces an immune mechanism that activates the mononuclear phagocyte system (MPS) cells in the myeloid tissues in the bone marrow, spleen, and skin tissue which then circulates and infiltrates many organs including the kidney (Machnik et al., 2009). These activated MPS cells release TNF‐α that appears in the circulation as its soluble form (sTNF‐α). sTNF‐α is formed by the proteolytic cleavage from membrane‐tethered form (mTNF‐α) by TNF‐α‐converting enzyme (TACE), a sheddase that is also identified as a disintegrin and metalloprotease 17 (ADAM17) (Black et al., 1997; Kriegler et al., 1988; Moss et al., 1997). Along with the production of TNF‐α, activated MPS cells also release abundant NO mostly from eNOS activation (Connelly et al., 2003). The critical observation of the differential TNF‐α responses in the present study indicates that chronic HS intake, on the one hand, increases sTNF‐α in the plasma, but reduces mTNF‐α in the renal tissue, on the other hand, most likely by increasing TACE activity (Black et al., 1997; Kriegler et al., 1988; Moss et al., 1997). Although it has been shown that DOCA‐salt hypertension was prevented by TACE knockdown in the brain (Xia et al., 2013), its exact regulation in the kidney during chronic HS intake has not been clearly defined. Further investigation would be needed to address this tissue. However, it can be added here that TNF‐α exerts its biological responses via interaction with two cell surface receptors, TNF‐α receptor type 1 (TNFR1) and type 2 (TNFR2), which are differentially expressed and regulated in the kidney (Al‐Lamki & Mayadas, 2015). While mTNF‐α has an equal affinity for both the receptors, the circulating sTNF‐α has the maximal high affinity to interact with TNFR1 with no or minimal affinity for TNFR2 (Grell et al., 1998). In the kidney, where TNFR2 plays a role in renal inflammation (Singh et al., 2013), TNFR1 activation induces a natriuretic effect by inhibiting tubular Na+ transportation and ENaC activity (Castillo et al., 2012) indicating a protective role for TNF‐α‐TNFR1 axis during chronic HS intake. In such a situation, the increases in plasma TNF‐α (sTNF‐α) in response to chronic HS intake observed in the present study would carry a more significant role in maintaining sodium homeostasis than the renal tissue changes in TNF‐α (mTNF‐α) levels. It is interesting to note that the chronic HS intake reduces the plasma level of IL‐6 in the present study (Figure 3a), indicating a differential role of these pro‐inflammatory cytokines in controlling sodium homeostasis. Therefore, it appears that while TNF‐α is released into the circulation by the activated mononuclear phagocyte system (MPS) through osmoregulatory mechanism during HS intake, the release of IL‐6 seems to mostly depend on other factors such as elevated renin‐angiotensin system (Chamarthi et al., 2011). During HS intake alone, the renin‐angiotensin system is downregulated, and thus, its impact on IL‐6 formation is reflected in both plasma and renal concentration of this cytokine. Further experiments would be required to understand more about the regulatory role of IL‐6 in renal excretory function.

In the present study, it is noted that eNOSKO mice given the NS diet have a higher degree of renal tubulointerstitial fibrosis, but not glomerular sclerosis, compared to WT mice. These changes seem to be minimized in eNOSKO mice given the HS diet, reflecting the effects of the reduced level of pro‐inflammatory cytokines (TNF‐α and IL‐6) in renal tissue. Such diminished renal injury findings in HS‐fed eNOSKO mice seem contradicted with the findings in another study in eNOSKO mice reported earlier (Daumerie et al., 2010) which did not measure the renal cytokine levels. However, this difference in the renal injury effects in that study (Daumerie et al., 2010) compared to the present study could be related to the age of mice (experiments starts at 6 months vs. 2 months age) as well as the high salt content (8% vs. 4% NaCl) in the diet given for a longer period (8 weeks vs. 2 weeks). The results in the present study indicate that, at least in the short‐term, HS intake suppressed the renal cytokine levels that help to minimize renal injury at the early stage.

The physiological or pathophysiological significance of these findings that HS intake for at least 2 weeks induces a reduction in the renal levels of pro‐inflammatory cytokines, may be important to explore further. Although pro‐inflammatory cytokine, particularly TNF‐α has been implicated in the development of salt‐sensitivity and hypertension (Majid et al., 2015; Rodríguez‐Iturbe et al., 2014; Schiffrin, 2013), earlier investigations in our laboratory (Shahid et al., 2008) demonstrate that TNF‐α administration induces the diuretic and natriuretic responses which indicate that TNF‐α plays a counter‐regulatory role in the mediation of salt‐sensitive hypertension. Thus, a reduction in the renal levels of pro‐inflammatory cytokines such as TNF‐α in response to HS intake for 2 weeks may enhance the tubular reabsorptive function that may facilitate sodium retention in the body. Such retention of sodium may be enhanced manifold in the condition of oxidative stress induced by NO deficiency. Salt‐sensitivity observed in eNOSKO mice could result from such enhanced sodium retention in the condition of oxidative stress as we have demonstrated earlier (Kopkan et al., 2010). Further studies may be required to understand the comprehensive mechanistic relationship between the reductions in renal levels of pro‐inflammatory cytokines and the induction of hypertension in response to chronic HS intake. However, it is reasonable to postulate that such decreases in renal levels of TNF‐α and other pro‐inflammatory cytokines facilitate sodium retention leading to the induction of hypertension in response to the intake of HS diet.

## 5 CONCLUSION

The findings in the present study demonstrate that HS intake, at least given for the 2‐week period, generally suppresses the renal levels of inflammatory cytokines in intact as well as in eNOS deficient condition. Overall, these data suggest that HS intake usually resulted in the downregulation of pro‐ and anti‐inflammatory cytokine in the kidney. Such downregulation of cytokine production, particularly TNF‐α during HS intake, may be critical in the condition of eNOS deficiency that may enhance the renal tubular sodium reabsorption leading to salt‐retention, and thus, facilitates the consequent development of salt‐sensitive hypertension.

## CONFLICT OF INTEREST

None.

## AUTHORS CONTRIBUTIONS

PS and DM designed the research project. PS, RS, and AC performed the experiments. PS and RS analyzed the data. PS and DM drafted, edited the manuscript. PS, RS, AC, and DM approved the final version of the manuscript.

## References

[phy214621-bib-0001] Al‐Lamki, R. S. , & Mayadas, T. N. (2015). TNF receptors: Signaling pathways and contribution to renal dysfunction. Kidney International, 87(2), 281–296. 10.1038/ki.2014.285 25140911

[phy214621-bib-0002] Black, R. A. , Rauch, C. T. , Kozlosky, C. J. , Peschon, J. J. , Slack, J. L. , Wolfson, M. F. , Castner, B. J. , Stocking, K. L. , Reddy, P. , Srinivasan, S. , Nelson, N. , Boiani, N. , Schooley, K. A. , Gerhart, M. , Davis, R. , Fitzner, J. N. , Johnson, R. S. , Paxton, R. J. , March, C. J. , & Cerretti, D. P. (1997). A metalloproteinase disintegrin that releases tumour‐necrosis factor‐alpha from cells. Nature, 385, 729–733. 9034190 10.1038/385729a0

[phy214621-bib-0003] Castillo, A. , Islam, M. T. , Prieto, M. C. , & Majid, D. S. (2012). Tumor necrosis factor‐α receptor type 1, not the type 2, mediates its acute responses in the kidney. The American Journal of Physiology‐Renal Physiology, 302, F1650–F1657. 22461305 10.1152/ajprenal.00426.2011 PMC3378101

[phy214621-bib-0004] Chamarthi, B. , Williams, G. H. , Ricchiuti, V. , Srikumar, N. , Hopkins, P. N. , Luther, J. M. , Jeunemaitre, X. , & Thomas, A. (2011). Inflammation and hypertension: The interplay of interleukin‐6, dietary sodium and the Renin‐Angiotensin system in humans. American Journal of Hypertension, 24(10), 1143–1148. 10.1038/ajh.2011.113 21716327 PMC3807212

[phy214621-bib-0005] Connelly, L. , Jacobs, A. T. , Palacios‐Callender, M. , Moncada, S. , & Hobbs, A. J. (2003). Macrophage endothelial nitric‐oxide synthase autoregulates cellular activation and pro‐inflammatory protein expression. Journal of Biological Chemistry, 278(29), 26480–26487. 10.1074/jbc.M302238200 12740377

[phy214621-bib-0006] Cook, S. , Vollenweider, P. , Ménard, B. , Egli, M. , Nicod, P. , & Scherrer, U. (2003). Increased eNO and pulmonary iNOS expression in eNOS null mice. European Respiratory Journal, 21(5), 770–773. 10.1183/09031936.03.00121203 12765418

[phy214621-bib-0007] Costa, A. P. , de Paula, R. C. , Carvalho, G. F. , Araújo, J. P. , Andrade, J. M. , de Almeida, O. L. , de Faria, E. C. , Freitas, W. M. , Coelho, O. R. , Ramires, J. A. , Quinaglia e Silva, J. C. , & Sposito, A. C. (2012). Brasilia Heart Study Group. High sodium intake adversely affects oxidative‐inflammatory response, cardiac remodelling and mortality after myocardial infarction. Atherosclerosis, 222, 284–291. 10.1016/j.atherosclerosis.2012.02.037 22436606

[phy214621-bib-0008] Das, U. N. (2006). Hypertension as a low‐grade systemic inflammatory condition that has its origins in the perinatal period. The Journal of the Association of Physicians of India, 54, 133–142. 16715619

[phy214621-bib-0009] Daumerie, G. , Bridges, L. K. , Yancey, S. , Davis, W. , Huang, P. , Loscalzo, J. , & Pointer, M. A. (2010). The effect of salt on renal damage in eNOS deficient mice. Hypertension Research, 33(2), 170–176. 10.1038/hr.2009.197 19960018 PMC4419703

[phy214621-bib-0010] Drenjančević‐Perić, I. , Jelaković, B. , Lombard, J. H. , Kunert, M. P. , Kibel, A. , & Grosa, M. (2011). High‐salt diet and hypertension: Focus on the Renin‐Angiotensin system. Kidney and Blood Pressure Research, 34(1), 1–11. 10.1159/000320387 21071956 PMC3214830

[phy214621-bib-0011] Francois, H. , Makhanova, N. , Ruiz, P. , Ellison, J. , Mao, L. , Rockman, H. A. , & Coffman, T. M. (2008). A role for the thromboxane receptor in L‐NAME hypertension. American Journal of Physiology‐Renal Physiology, 295, F1096–F1102. 18684890 10.1152/ajprenal.00369.2007 PMC2576155

[phy214621-bib-0012] Funakoshi, Y. , Ichiki, T. , Ito, K. , & Takeshita, A. (1999). Induction of interleukin‐6 expression by angiotensin II in rat vascular smooth muscle cells. Hypertension, 34, 118–125. 10406834 10.1161/01.hyp.34.1.118

[phy214621-bib-0013] Gijsbers, L. , Dower, J. I. , Schalkwijk, C. G. , Kusters, Y. H. , Bakker, S. J. , Hollman, P. C. , & Geleijnse, J. M. (2015). Effects of sodium and potassium supplementation on endothelial function and inflammation in untreated (pre)hypertensives: A fully controlled dietary intervention study. Journal of Hypertension, 33, e72. 10.1017/S0007114515002986 26343780

[phy214621-bib-0014] Gomori, G. (1950). A rapid one‐step trichrome stain. The American Journal of Clinical Pathology, 20, 661–664. 15432364 10.1093/ajcp/20.7_ts.661

[phy214621-bib-0015] Grell, M. , Wajant, H. , Zimmermann, G. , & Scheurich, P. (1998). The type 1 receptor (CD120a) is the high‐affinity receptor for soluble tumor necrosis factor. Proceedings of the National Academy of Sciences of the United States of America, 95, 570–575. 9435233 10.1073/pnas.95.2.570 PMC18461

[phy214621-bib-0016] Guzik, T. J. , Hoch, N. E. , Brown, K. A. , McCann, L. A. , Rahman, A. , Dikalov, S. , Goronzy, J. , Weyand, C. , & Harrison, D. G. (2007). Role of the T cell in the genesis of angiotensin II induced hypertension and vascular dysfunction. Journal of Experimental Medicine, 204, 2449–2460. 10.1084/jem.20070657 PMC2118469 17875676

[phy214621-bib-0017] Han, Y. , Runge, M. S. , & Braiser, A. R. (1999). Angiotensin II induces interleukin‐6 transcription in vascular smooth muscle cells through pleiotropic activation of nuclear factor‐κB transcription factors. Circulation Research, 84, 695–703. 10189357 10.1161/01.res.84.6.695

[phy214621-bib-0018] Harrison, D. G. (1997). Role of nitric oxide in inflammatory diseases. Cellular and molecular mechanisms of endothelial cell dysfunction. The Journal of Clinical Investigation, 100, 2153–2157. 9410891 10.1172/JCI119751 PMC508409

[phy214621-bib-0019] Islam, M. T. , Agarwal, D. , Francis, J. , Matrougui, K. , & Majid, D. S. A. (2011). Inhibition of nitric oxide synthase enhances the production of tumor necrosis factor‐alpha in macrophage cells. FASEB Journal, 25, A1030.

[phy214621-bib-0020] Kopkan, L. , Hess, A. , Husková, Z. , Červenka, L. , Navar, L. G. , & Majid, D. S. A. (2010). High‐salt intake enhances superoxide activity in eNOS knockout mice leading to the development of salt sensitivity. The American Journal of Physiology‐Renal Physiology, 299, F656–F663. 20610532 10.1152/ajprenal.00047.2010 PMC2944295

[phy214621-bib-0021] Kopkan, L. , & Majid, D. S. (2005). Superoxide contributes to development of salt sensitivity and hypertension induced by nitric oxide deficiency. Hypertension, 46(4), 1026–1031. 10.1161/01.HYP.0000174989.39003.58 16103275

[phy214621-bib-0022] Kramer, S. M. , Aggarwal, B. B. , Eessalu, T. E. , McCabe, S. M. , Ferraiolo, B. L. , Figari, I. S. , & Palladino, M. A. Jr (1988). Characterization of the in vitro and in vivo species preference of human and murine tumor necrosis factor‐alpha. Cancer Research, 48, 920–925. 2827889

[phy214621-bib-0023] Kriegler, M. , Perez, C. , DeFay, K. , Albert, I. , & Lu, S. D. (1988). A novel form of TNF/cachectin is a cell surface cytotoxic transmembrane protein: Ramifications for the complex physiology of TNF. Cell, 53(1), 45–53. 10.1016/0092-8674(88)90486-2 3349526

[phy214621-bib-0024] Lara, L. S. , McCormack, M. , Semprum‐Prieto, L. C. , Shenouda, S. , Majid, D. S. , Kobori, H. , Navar, L. G. , & Prieto, M. C. (2012). AT1 receptor‐mediated augmentation of angiotensinogen, oxidative stress, and inflammation in ANG II‐salt hypertension. The American Journal of Physiology‐Renal Physiology, 302, F85–F94. 21900456 10.1152/ajprenal.00351.2011 PMC3251338

[phy214621-bib-0025] Lee, B. H. , Yang, A. R. , Kim, M. Y. , McCurdy, S. , & Boisvert, W. A. (2017). Natural sea salt consumption confers protection against hypertension and kidney damage in Dahl salt‐sensitive rats. Food & Nutrition Research, 61(1), 1264713 10.1080/16546628.2017.1264713 28325999 PMC5328355

[phy214621-bib-0026] Liu, F. , Mu, J. , Yuan, Z. , Wu, G. , Liu, E. , Zheng, S. , Lian, Q. , Ren, K. , & Xu, H. (2012). High salt intake fails to enhance plasma adiponectin in normotensive salt‐sensitive subjects. Nutrition, 28(4), 422–425. 10.1016/j.nut.2011.08.018 22189196

[phy214621-bib-0027] Liu, V. W. , & Huang, P. L. (2008). Cardiovascular roles of nitric oxide: A review of insights from nitric oxide synthase gene disrupted mice. Cardiovascular Research, 77, 19–29. 17658499 10.1016/j.cardiores.2007.06.024 PMC2731989

[phy214621-bib-0028] Luna, L. G. (1992). Histopathological methods and color atlas of special stains and tissue artifacts, 1st ed. Gaitheresburg, MD: American Histolabs.

[phy214621-bib-0029] Luo, T. , Ji, W. J. , Yuan, F. , Guo, Z. Z. , Li, Y. X. , Dong, Y. , Ma, Y. Q. , Zhou, X. , & Li, Y. M. (2016). Th17/Treg imbalance induced by dietary salt variation indicates inflammation of target organs in humans. Scientific Reports, 6(1), 26767 10.1038/srep26767 27353721 PMC4926124

[phy214621-bib-0030] Machnik, A. , Neuhofer, W. , Jantsch, J. , Dahlmann, A. , Tammela, T. , Machura, K. , Park, J. K. , Beck, F. X. , Müller, D. N. , Derer, W. , Goss, J. , Ziomber, A. , Dietsch, P. , Wagner, H. , van Rooijen, N. , Kurtz, A. , Hilgers, K. F. , Alitalo, K. , Eckardt, K. U. , … Titze, J. (2009). Macrophages regulate salt‐dependent volume and blood pressure by a vascular endothelial growth factor‐C‐dependent buffering mechanism. Nature Medicine, 15(5), 545–552. 10.1038/nm.1960 19412173

[phy214621-bib-0031] Majid, D. S. A. , Prieto, M. C. , & Castillo, A. A. (2017). Chronic treatment with an inhibitor of nitric oxide synthase reduces protein expression of tumor necrosis factor‐alpha receptor type 1 in renal cortical tissues in mice. FASEB Journal, 31, A103.

[phy214621-bib-0032] Majid, D. S. A. , Prieto, M. C. , Chamberlain, C. M. , & Castillo, A. (2018). Chronic high salt intake reduces protein expression of tumor necrosis factor‐alpha receptor type 1 in renal cortical tissue of mice lacking the gene for endothelial nitric oxide synthase. FASEB Journal. 32, A751.5

[phy214621-bib-0033] Majid, D. S. A. , Prieto, M. C. , & Navar, L. G. (2015). Salt‐sensitive hypertension: Perspectives on intrarenal mechanisms. Current Hypertension Reviews, 11, 38–48. 26028244 10.2174/1573402111666150530203858 PMC4626881

[phy214621-bib-0034] Mallamaci, F. , Leonardis, D. , Pizzini, P. , Cutrupi, S. , Tripepi, G. , & Zoccali, C. (2013). Procalcitonin and the inflammatory response to salt in essential hypertension: A randomized cross‐over clinical trial. Journal of Hypertension, 31, 1424–1430. 23743803 10.1097/HJH.0b013e328360ddd5

[phy214621-bib-0035] Mattson, D. L. (2014). Infiltrating immune cells in the kidney in salt‐sensitive hypertension and renal injury. The American Journal of Physiology‐Renal Physiology, 307, F499–F508. 25007871 10.1152/ajprenal.00258.2014 PMC4154114

[phy214621-bib-0036] Mattson, D. L. , & Higgins, D. J. (1996). Influence of dietary sodium intake on renal medullary nitric oxide synthase. Hypertension, 27, 688–692. 8613226 10.1161/01.hyp.27.3.688

[phy214621-bib-0037] Moncada, S. , Palmer, R. M. , & Higgs, E. A. (1991). Nitric oxide: Physiology, pathophysiology, and pharmacology. Pharmacological Reviews, 43, 109–142. 1852778

[phy214621-bib-0038] Moss, M. L. , Jin, S. L. , Milla, M. E. , Bickett, D. M. , Burkhart, W. , Carter, H. L. , Chen, W. J. , Clay, W. C. , Didsbury, J. R. , Hassler, D. , Hoffman, C. R. , Kost, T. A. , Lambert, M. H. , Leesnitzer, M. A. , McCauley, P. , McGeehan, G. , Mitchell, J. , Moyer, M. , Pahel, G. , … Becherer, J. D. (1997). Cloning of a disintegrin metalloproteinase that processes precursor tumour‐necrosis factor‐alpha. Nature, 385, 733–736. 9034191 10.1038/385733a0

[phy214621-bib-0039] Nakamura, T. , Kataoka, K. , Tokutomi, Y. , Nako, H. , Toyama, K. , Dong, Y. F. , Koibuchi, N. , Yamamoto, E. , Yasuda, O. , Ogawa, H. , & Kim‐Mitsuyama, S. (2011). Novel mechanism of salt‐induced glomerular injury: Critical role of eNOS and angiotensin II. Journal of Hypertension, 29, 1528–1535. 21720272 10.1097/HJH.0b013e328348ca95

[phy214621-bib-0040] Numanami, H. , Nelson, D. K. , Hoyt, J. C. , Freels, J. L. , Habib, M. , Amano, J. , Haniuda, M. , Koyama, S. , & Robbins, R. A. (2003). Peroxynitrite enhances interleukin‐10 reduction in the release of neutrophil chemotactic activity. The American Journal of Respiratory Cell and Molecular Biology, 29, 239–244. 12626343 10.1165/rcmb.2002-0275OC

[phy214621-bib-0041] Rodríguez‐Iturbe, B. , Pons, H. , Quiroz, Y. , & Johnson, R. J. (2014). The immunological basis of hypertension. American Journal of Hypertension, 27(11), 1327–1337. 10.1093/ajh/hpu142 25150828 PMC4263946

[phy214621-bib-0042] Rodriguez‐Iturbe, B. , Vaziri, N. D. , Herrera‐Acosta, J. , & Johnson, R. J. (2004). Oxidative stress, renal infiltration of immune cells, and salt‐sensitive hypertension: All for one and one for all. American Journal of Physiology‐Renal Physiology, 286(4), F606–F616. 10.1152/ajprenal.00269.2003 15001451

[phy214621-bib-0043] Ruiz‐Ortega, M. , Ruperez, M. , Lorenzo, O. , Esteban, V. , Blanco, J. , Mezzano, S. , & Egido, J. (2002). Angiotensin II regulates the synthesis of proinflammatory cytokines and chemokines in the kidney. Kidney International, 82, S12–S22. 10.1046/j.1523-1755.62.s82.4.x 12410849

[phy214621-bib-0044] Sanz‐Rosa, D. , Oubina, M. P. , Cediel, E. , De Las Heras, N. , Vegazo, O. , Jimenez, J. , Lahera, V. , & Cachofeiro, V. (2005). Effect of AT1 receptor antagonism on vascular and circulating inflammatory mediators in SHR: Role of NFκB/IκB system. The American Journal of Physiology: Heart and Circulatory Physiology, 288, H111–H115. 15308481 10.1152/ajpheart.01061.2003

[phy214621-bib-0045] Schiffrin, E. L. (2013). The immune system: Role in hypertension. Canadian Journal of Cardiology, 29(5), 543–548. 10.1016/j.cjca.2012.06.009 22902155

[phy214621-bib-0046] Shahid, M. , Francis, J. , & Majid, D. S. (2008). Tumor necrosis factor‐ induces renal vasoconstriction as well as natriuresis in mice. The American Journal of Physiology‐Renal Physiology, 295, F1836–F1844. 18922887 10.1152/ajprenal.90297.2008 PMC2604828

[phy214621-bib-0047] Shahid, M. , Francis, J. , Matrougui, K. , & Majid, D. S. (2010). Involvement of tumor necrosis factor‐alpha in natriuretic response to systemic infusion of nitric oxide synthase inhibitor in anesthetized mice. The American Journal of Physiology‐Renal Physiology, 299, F217–F224. 20410217 10.1152/ajprenal.00611.2009 PMC2904181

[phy214621-bib-0048] Singh, P. , Bahrami, L. , Castillo, A. , & Majid, D. S. (2013). TNF‐α type 2 receptor mediates renal inflammatory response to chronic angiotensin II administration with high salt intake in mice. American Journal of Physiology‐Renal Physiology, 304(7), F991–F999. 10.1152/ajprenal.00525.2012 23389459 PMC3625853

[phy214621-bib-0049] Singh, P. , Castillo, A. , Islam, M. T. , & Majid, D. S. A. (2017). Evidence for prohypertensive, proinflammatory effect of interleukin‐10 during chronic high salt intake in the condition of elevated Angiotensin II level. Hypertension, 70(4), 839–845. 10.1161/HYPERTENSIONAHA.117.09401 28847894 PMC5657538

[phy214621-bib-0050] Singh, P. , Castillo, A. , & Majid, D. S. (2014). Decrease in IL‐10 and increase in TNF‐α levels in renal tissues during systemic inhibition of nitric oxide in anesthetized mice. Physiological Reports, 2, e00228. 24744897 10.1002/phy2.228 PMC3966239

[phy214621-bib-0051] Xia, H. , Sriramula, S. , Chhabra, K. H. , & Lazartigues, E. (2013). Brain angiotensin‐converting enzyme type 2 shedding contributes to the development of neurogenic hypertension. Circulation Research, 113(9), 1087–1096. 10.1161/CIRCRESAHA.113.301811 24014829 PMC4479408

[phy214621-bib-0052] Yilmaz, R. , Akoglu, H. , Altun, B. , Yildirim, T. , Arici, M. , & Erdem, Y. (2012). Dietary salt intake is related to inflammation and albuminuria in primary hypertensive patients. European Journal of Clinical Nutrition, 66(11), 1214–1218. 10.1038/ejcn.2012.110 22909578

[phy214621-bib-0053] Zhu, H. , Pollock, N. K. , Kotak, I. , Gutin, B. , Wang, X. , Bhagatwala, J. , Parikh, S. , Harshfield, G. A. , & Dong, Y. (2014). Dietary sodium, adiposity, and inflammation in healthy adolescents. Pediatrics, 133, e635–e642. 10.1542/peds.2013-1794 24488738 PMC3934330

